# Rapid emergence and predominance of a broadly recognizing and fast-evolving norovirus GII.17 variant in late 2014

**DOI:** 10.1038/ncomms10061

**Published:** 2015-12-02

**Authors:** Martin C. W. Chan, Nelson Lee, Tin-Nok Hung, Kirsty Kwok, Kelton Cheung, Edith K. Y. Tin, Raymond W. M. Lai, E. Anthony S. Nelson, Ting F. Leung, Paul K. S. Chan

**Affiliations:** 1 Department of Microbiology, Faculty of Medicine, 1/F Lui Che Woo Clinical Sciences Building, Prince of Wales Hospital, Chinese University of Hong Kong, Hong Kong, China; 2 Department of Medicine and Therapeutics, Faculty of Medicine, 9/F Lui Che Woo Clinical Sciences Building, Prince of Wales Hospital, Chinese University of Hong Kong, Hong Kong, China; 3 Department of Paediatrics, Faculty of Medicine, 6/F Lui Che Woo Clinical Sciences Building, Prince of Wales Hospital, Chinese University of Hong Kong, Hong Kong, China

## Abstract

Norovirus genogroup II genotype 4 (GII.4) has been the predominant cause of viral gastroenteritis since 1996. Here we show that during the winter of 2014–2015, an emergent variant of a previously rare norovirus GII.17 genotype, Kawasaki 2014, predominated in Hong Kong and outcompeted contemporary GII.4 Sydney 2012 in hospitalized cases. GII.17 cases were significantly older than GII.4 cases. Root-to-tip and Bayesian BEAST analyses estimate GII.17 viral protein 1 (VP1) evolves one order of magnitude faster than GII.4 VP1. Residue substitutions and insertion occur in four of five inferred antigenic epitopes, suggesting immune evasion. Sequential GII.4-GII.17 infections are noted, implicating a lack of cross-protection. Virus bound to saliva of secretor histo-blood groups A, B and O, indicating broad susceptibility. This fast-evolving, broadly recognizing and probably immune-escaped emergent GII.17 variant causes severe gastroenteritis and hospitalization across all age groups, including populations who were previously less vulnerable to GII.4 variants; therefore, the global spread of GII.17 Kawasaki 2014 needs to be monitored.

## Abstract (web summary)

Norovirus GII.4 has been predominating in viral gastroenteritis for 20 years. Here the authors report the emergence and predominance of a novel fast-evolving GII.17 lineage norovirus causing viral gastroenteritis in Hong Kong, with a shift in age distribution of affected individuals towards an older age as compared to GII.4.

## Graphical abstract

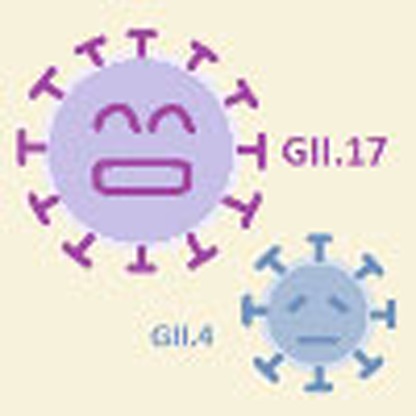

Human norovirus, of the genus *Norovirus* and family *Caliciviridae,* is the leading cause of acute gastroenteritis worldwide^1^. Genetically, this virus is classified into at least 6 genogroups and nearly 40 genotypes based on viral protein 1 (VP1) sequences^2^. Despite the broad genetic diversity, one genotype, known as genogroup II genotype 4 (GII.4), has been the major circulating genotype in community and health-care settings since 1996 with periodic emergence of novel GII.4 variants every 2–4 years^3^. Norovirus GII.4 infections are associated with more severe clinical manifestations and hospitalization^4^. Our earlier hospital-based study also indicated that GII.4 accounted for nearly 90% of hospitalized norovirus gastroenteritis that represents the severe end of the spectrum of disease^5^. Despite the overwhelmingly predominance of norovirus GII.4 strains in the past two decades, studies on historical stool specimens dated back to the 1970s and 1980s suggested that non-GII.4 norovirus such as GII.3 was commonly detected, at least in hospitalized paediatric diarrhoeal cases^6^. This raises the possibility that epidemic norovirus may not be limited only to the GII.4 genotype. Notably, accumulating evidence suggests that the circulation of different norovirus genotypes is highly dynamic, probably as a result of herd immunity^7^.

In late 2014, we observe a rapid increase in the number of hospitalized norovirus gastroenteritis attributed to the emergent GII.17 Kawasaki 2014 in Hong Kong. Here, we report epidemiological and virological characterization of norovirus GII.17 cases. Our findings suggest close monitoring of the global spread of this emergent GII.17 Kawasaki 2014 is necessary.

## Results

### High norovirus GII.17 activity during 2014–2015 winter

**Figure 1:**
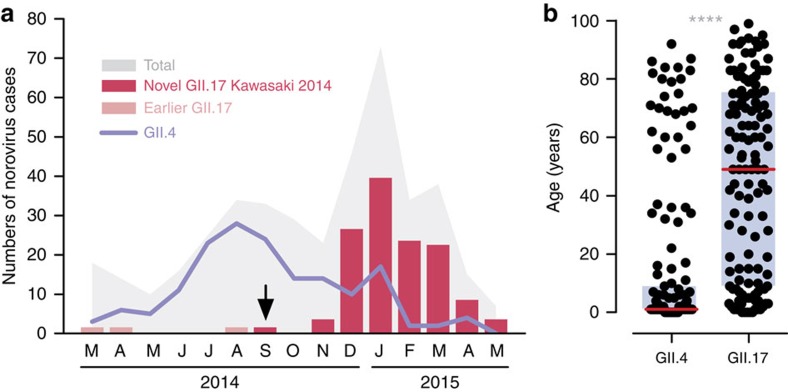
High activity of norovirus GII.17 during the 2014–2015 winter in Hong Kong. (**a**) Temporal distribution of hospitalized norovirus GII.4 and GII.17 cases during the study period from March 2014 to May 2015. Black arrow denotes first detection (27 September 2014) of the novel GII.17 Kawasaki 2014. (**b**) Age distribution of norovirus GII.4 (*n*=163) and GII.17 (*n*=128) cases. Red horizontal lines and purple rectangular shades indicate medians and interquartile ranges, respectively. ****P<0.0001 (Mann–Whitney *U-*test).

A total of 415 laboratory-confirmed hospitalized norovirus cases were studied in our ongoing local norovirus surveillance between March 2014 and May 2015, inclusively. The median age of patients was 6 years (interquartile range (IQR): 1–58 years). The male-to-female ratio was 1.14. Typical winter seasonality with higher norovirus activity in winter months (December 2014 to March 2015) was observed (Fig. 1a). Norovirus genotype was successfully identified in 372 (89.6%) cases. During the inter-seasonal period (March 2014–November 2014), the predominant circulating norovirus genotype was GII.4 (all but 2 belonged to the Sydney 2012) that accounted for 69.2% (128/185) of genotyped cases; GII.17 cases were only rarely (3.8%; 7/185) detected (Fig. 1a). A rapid rise in the number and proportion of GII.17 cases was observed in winter months during which GII.17 cases predominated over GII.4 cases (65.9% (110/167) versus 18.6% (31/167); *P*<0.0001, Fisher's exact test; Fig. 1a). Overall, the three most common norovirus genotypes detected during the report period were GII.4 (43.8%; *n*=163), GII.17 (34.4%; *n*=128) and GII.6 (8.9%; *n*=33).

### Wide age distribution of norovirus GII.17 infections

The median (IQR) age of GII.17 (*n*=128) cases was significantly older than that of GII.4 (*n*=163) cases (49 (9–75) versus 1 (1–8) years; *P*<0.0001, Mann–Whitney *U*-test; Fig. 1b). By age group, the proportion of children (aged <5 years), older children and young adults (aged 5–65 years) and older adults (aged >65 years) in GII.17 cases were 15.6% (20/128), 47.7% (61/128) and 36.7% (47/128), respectively (GII.4: 70.0% (114/163), 19.0% (31/163) and 11.0% (18/163), respectively). Elderly aged ≥85 years were more commonly observed in GII.17 cases than in GII.4 cases (11.7% (15/128) versus 1.8% (3/163); *P*<0.001, Fisher's exact test). In adults, proportion of cases with major underlying immunocompromising comorbidities (for example, malignancy and organ transplantation) were comparable in GII.17 and GII.4 cases (14% versus 12%; *P*>0.99, Fisher's exact test). One GII.17 case required admission to an intensive care unit and two other GII.17 cases had fatal outcomes; none were reported in GII.4 cases.

### High norovirus GII.17 activity with an emergent variant

**Figure 2:**
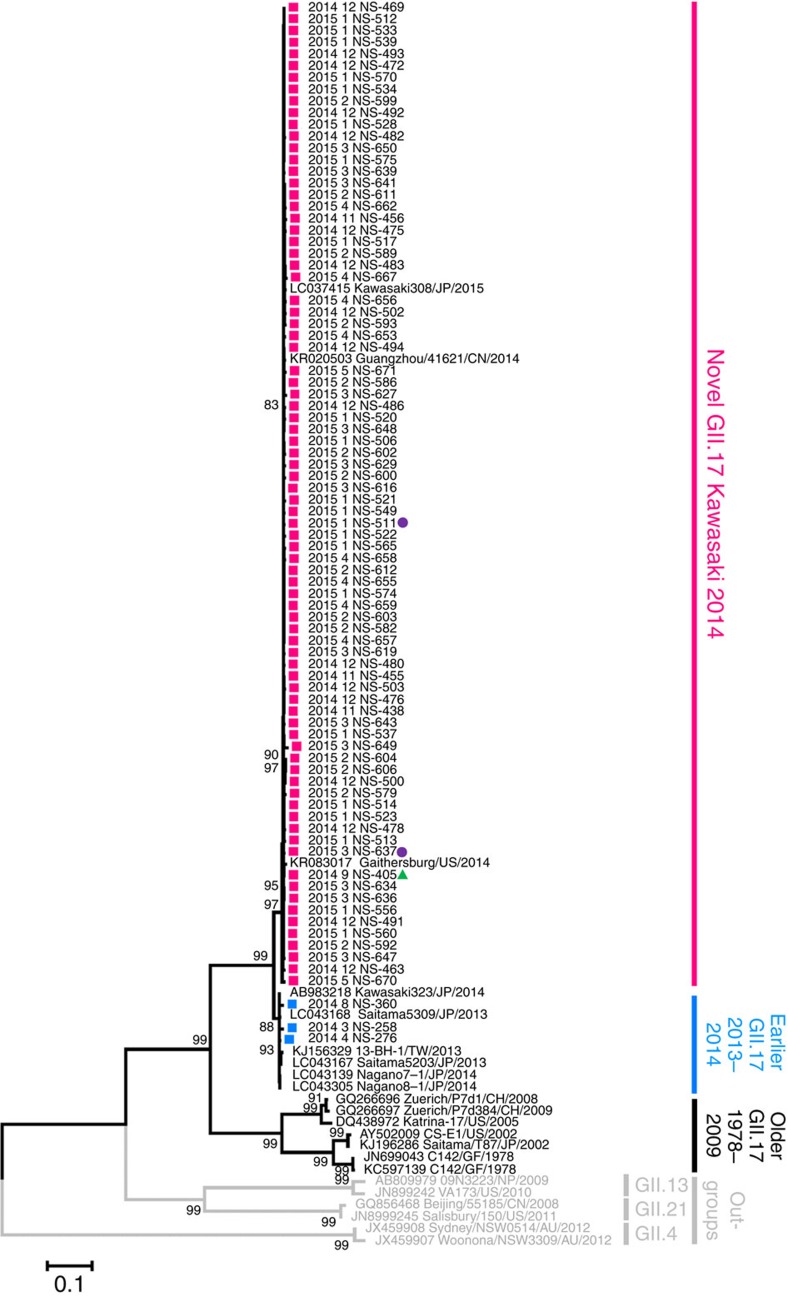
Emergence of norovirus GII.17 Kawasaki 2014 during the 2014–2015 winter in Hong Kong. Maximum likelihood phylogenetic inference of complete and near complete VP1 nucleotide sequences was performed. Gaps in alignment were neglected in the analysis and the final data set contained 1,586 nucleotide positions. Shown are trees with the highest log likelihood. Statistical evaluation was performed by 1,000 bootstrap replications and percentages of clustering (≥80%) are shown at nodes. Magenta and blue squares denote GII.17 Kawasaki 2014 and earlier GII.17 sequences obtained in this study, respectively. Green triangle denotes the first case of GII.17 Kawasaki 2014 in Hong Kong. Purple circles indicate cases selected for saliva-binding assay. Scale bars indicate the number of substitutions per site. Sequence nomenclature: year–month of collection followed by unique sequence identifier (for GII.17 sequences acquired in this study); GenBank accession number followed by unique sequence identifier, country of origin and year of collection (for GII.17 sequences downloaded from GenBank). Country/region abbreviations: AU, Australia; CH, Switzerland; CN, China; GF, French Guiana; JP, Japan; NP, Nepal; TW, Taiwan; US, the United States of America.

Complete VP1 sequences were determined in a subset of 84 (65.6%) out of 128 GII.17 cases detected during the study period (Supplementary Table 1). At least ten (or all if less than ten were available) GII.17 cases per month were randomly selected and sequenced. Maximum likelihood phylogenetic inference of VP1 nucleotide sequences rooted to non-GII.17 genotypes showed that all GII.17 cases collected on or before August 2014 clustered together with contemporary GII.17 strains in Japan (Kawasaki323/2014) and Taiwan (13-BH-1/2013; Fig. 2). From September 2014 onwards, however, all GII.17 sequences grouped to a distant, separate cluster that included other recently reported GII.17 associated with increased activity during the 2014–2015 winter in China (Guangzhou/41621/2014) and Japan (Kawasaki308/2015; Fig. 2). Identical clustering was observed using complete VP1 amino-acid sequences (Supplementary Fig. 1a). Identical clustering was also observed using nucleotide and amino-acid trees rooted to the oldest GII.17 strains but discordant tree topology to the rooting by non-GII.17 outgroups was noted (Supplementary Fig. 1b). These most recent GII.17 cases showed 4.3–5.0% pairwise amino-acid difference in VP1 from the phylogenetically closest GII.17 sequences collected from Japan and Taiwan in 2013–2014. This suggested a new GII.17 variant adapting the same criterion for defining a new GII.4 variant^8^. This novel GII.17 variant was named ‘Kawasaki 2014' by the NoroNet^9^.

### Genetic signatures in HBGA binding and antigenic epitopes

**Figure 3:**
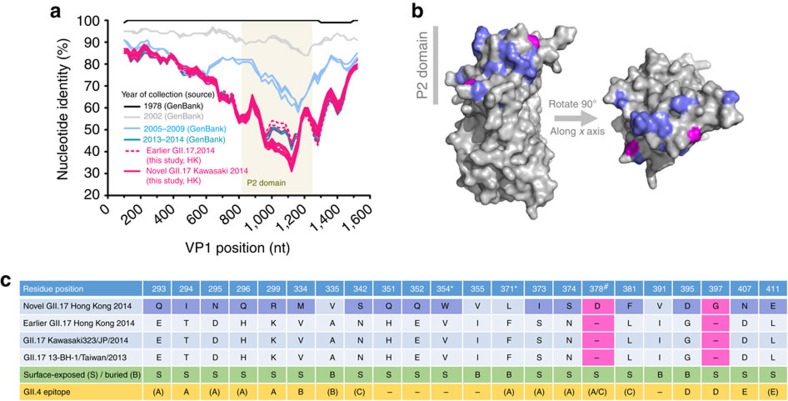
Genetic signatures in inferred antigenic epitopes of norovirus GII.17 Kawasaki 2014. (**a**) Time-ordered SimPlot analysis of all complete and near complete norovirus GII.17 VP1 nucleotide sequences obtained in this study and GenBank. (**b**) Three-dimensional VP1 structure of the novel GII.17 Kawasaki 2014 predicted by homologous modelling based on the crystal structure of a GII.10 strain (PDB number 3V7A). Surface-exposed mutations and insertions, compared with other earlier local and regional GII.17 VP1 sequences collected in 2013–2014, are coloured in purple and magenta, respectively. (**c**) List of amino-acid changes in the P2 domain of the novel GII.17 Kawasaki 2014 compared with other earlier local and regional GII.17 VP1 sequences collected in 2013–2014. Surface-exposed mutations and insertions are highlighted as in **b**. Asterisk denotes residue under episodic diversifying (positive) selection. Number sign denotes residue insertion in the histo-blood group antigens binding interface. Parentheses refer to ‘in the vicinity of' which is defined as 1 to 2 residues adjacent to antigenic epitopes inferred from those of GII.4 using multiple sequence alignment^60^. Residues are numbered with reference to the novel GII.17 Kawasaki 2014. JP, Japan; nt, nucleotide.

Time-ordered SimPlot analysis of all complete and near complete norovirus GII.17 VP1 nucleotide sequences obtained in this study and GenBank indicated that the P2 domain was the most hypervariable region and underwent continuous evolution (Fig. 3a). The novel GII.17 Kawasaki 2014 showed the greatest divergence (30% identity at trough) in P2 domain from the oldest GII.17 VP1 sequence available (Hu/GII.17/C142/GF/1978, accession number KC597139; Fig. 3a). Compared with other earlier local and regional GII.17 VP1 sequences collected in 2013–2014, the novel GII.17 Kawasaki 2014 shared very high pairwise nucleotide identity (97.0–98.1%) in the shell and P1 domains but had substantially lower identity (90.6–92.7%) in the P2 domain. This translated to 22 amino-acid changes in the P2 domain, including 2 insertions at positions 378 and 397 (novel GII.17 Kawasaki 2014 numbering); 18 (82%) of the changes, including the 2 insertions, were surface exposed (Fig. 3b,c). Notably, the insertion at position 378 corresponds to D374 of GII.4 VP1 that comprised histo-blood group antigen (HBGA)-binding interface of various norovirus GII (ref. 10). Multiple alignment of GII.17 with GII.4 VP1 sequences suggested that 6 (positions 294, 299, 334, 395, 397 and 407) and 11 (positions 293, 295, 296, 335, 342, 371, 373, 374, 378, 381 and 411) of these amino-acid changes were mapped to and adjacent to inferred antigenic epitopes, respectively (Fig. 3c and Supplementary Fig. 2). Conservative mix-effect model of evolution (MEME) analysis inferred that two codons of GII.17 VP1 were under episodic diversifying (positive) selection (Table 1). Both codons (354 and 371; novel GII.17 Kawasaki 2014 numbering) resided in the P2 domain but were neither mapped to or in the vicinity of any inferred antigenic epitopes. Among the 81 sequenced complete VP1 of the novel GII.17 Kawasaki 2014, there were 144 single-nucleotide polymorphisms of which 19 were non-synonymous. Six of the non-synonymous single-nucleotide polymorphisms resided in the P2 domain.

### Accelerated evolution of norovirus GII.17 VP1 since 2002

**Figure 4:**
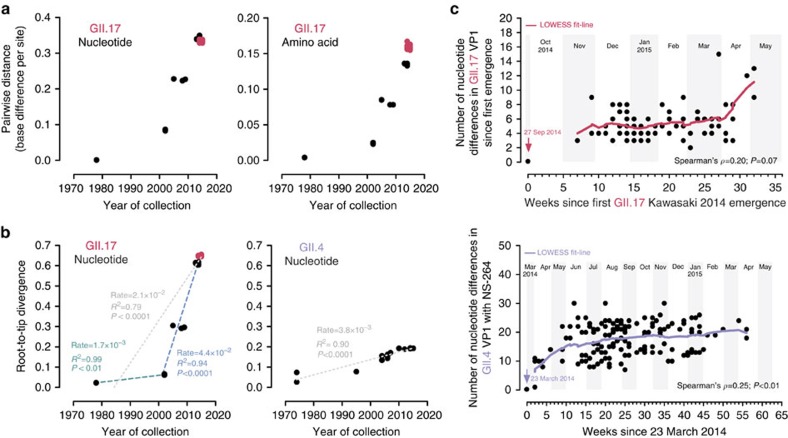
Rapid evolution of norovirus GII.17 VP1. (**a**) Pairwise GII.17 VP1 nucleotide and amino-acid distance comparison. (**b**) Root-to-tip regression analysis to estimate GII.17 VP1 nucleotide substitution rates without a molecular clock assumption. Magenta circles indicate the novel GII.17 Kawasaki 2014. Green and blue dotted lines denote best fit lines for GII.17 from 1978 to 2002 and 2002 to 2015, respectively. Grey dotted lines denote best fit lines for both GII.4 and GII.17 from 1970s to 2015. Substitution rates are expressed as the number of nucleotide substitutions per site per year. Complete and near complete GII.17 VP1 sequences were used. GII.4 VP1 sequences used: CHDC5191/USA/1974 (GenBank accession number: JX023286; variant: ancestral); CHDC2094/USA/1974 (FJ537135; ancestral); calicivirus/USA/1995 (AJ004864; US-95/96); Farmington Hills/USA/2004 (JQ478408; Famington Hills 2002); Hunter 284E/AU/2004 (DQ078794; Hunter 2004); Yerseke38/NL/2006 (EF126963; Yerseke 2006); Sakai2/JP/2006 (AB447448; Asia 2003); Kumanoto4/JP/2006 (AB447462; Den Haag 2006); Apeldoorn317/NL/2007 (AB445395; Apeldoorn 2007); New Orleans/USA/2010 (JN595867; New Orleans 2009); NSW0514/AU/2012 (JX459908; Sydney 2012); CUHK-NS-264/HKG/2014 (JX459908; Sydney 2012); CUHK-NS-508/HKG/2015 (KT780371; Sydney 2012). (**c**) Upper panel. Number of accumulated VP1 nucleotide differences of local GII.17 Kawasaki 2014 compared with the first strain (magenta arrow) detected on 27 September 2014 in Hong Kong against time (in weeks) since first emergence. Lower panel. Number of accumulated VP1 nucleotide differences of local GII.4 Sydney 2012 collected between March 2014 and May 2015 compared with the earliest local strain NS-264 (purple arrow) collected on 23 March 2014 during the study period. Magenta and purple lines denote the LOWESS fit-lines for GII.17 Kawasaki 2014 and GII4 Sydney 2012, respectively. The earliest local GII.17 Kawasaki 2014 (NS-405) and GII.4 Sydney 2012 (NS-264) strains were excluded in LOWESS data fitting and Spearman's correlation calculation.

Pairwise nucleotide and amino-acid distance comparison of all complete and near complete norovirus GII.17 VP1 sequences obtained in this study and GenBank with reference to the oldest complete GII.17 VP1 sequence available (Hu/GII.17/C142/GF/1978) indicated slower evolution of GII.17 VP1 from 1978 to 2002 but an accelerated evolution from 2002 to 2015 (Fig. 4a). The difference was more prominent on the amino-acid level. Nucleotide divergence rate estimation using root-to-tip regression analysis without a molecular clock assumption revealed a similar two-phase evolution of GII.17 VP1 (Fig. 4b). The divergence rate from 1978 to 2002 and 2002 to 2015 was 1.7 × 10^−3^ and 4.4 × 10^−2^ nucleotide substitutions per site per year, respectively; the combined rate was 2.1 × 10^−2^ nucleotide substitutions per site per year. Using representative local and GenBank GII.4 VP1 sequences, the divergence rate of GII.4 VP1 from 1974 to 2015 was 3.8 × 10^−3^ nucleotide substitutions per site per year (Fig. 4b), which was comparable in magnitude to previous estimations (4.3 × 10^−3^−7.2 × 10^−3^ nucleotide substitutions per site per year)^11^,^12^,^13^. Using Bayesian uncorrelated lognormal relaxed molecular clock model, GII.17 VP1 was estimated to evolve since 1978 at a rate of 1.2 × 10^−2^ (95% highest posterior density interval: 1.1 × 10^−3^−4.0 × 10^−2^) nucleotide substitutions per site per year.

### Rapid evolution of norovirus GII.17 after epidemic spread

A total of 81 complete VP1 sequences of GII.17 Kawasaki 2014 were determined. Pairwise nucleotide comparison with the first strain (the prototype NS-405 collected on 27 September 2014) suggested an overall increase in differences over time (*ρ*=0.20; *P*=0.07, Spearman's test; Fig. 4c, upper panel). Locally weighted scatterplot smoothing (LOWESS) data fitting narrowed down rapid virus evolution to a period after late March/early April 2015 when the norovirus activity was subsiding. During the 36-week period since first emergence of GII.17 Kawasaki 2014, a maximum number of 15 nucleotide differences was observed against the prototype; this was equivalent to an average divergence rate of 1.3 × 10^−2^ nucleotide substitutions per site per year. For comparison, 155 complete VP1 sequences of GII.4 Sydney 2012 collected between March 2014 and May 2015 were determined (Supplementary Table 2). Pairwise nucleotide comparison with the earliest local strain NS-264 (collected on 23 March 2014) during the study period revealed an overall increase in differences over time (*ρ*=0.25; *P*<0.01, Spearman's test; Fig. 4c, lower panel). However, LOWESS data fitting suggested local GII.4 Sydney 2012 strains remained relatively stable on genetic level during the emergence and predominance of GII.17 Kawasaki 2014 in late 2014 and throughout 2015 (January–April).

### Complete genome analysis of norovirus GII.17

Complete/near complete genomes were determined on 27 GII.17 Kawasaki 2014 cases collected at different months (Supplementary Table 1). One representative consensus GII.17 Kawasaki 2014 strain collected at the peak month (December 2014) shared highest nucleotide identity to earlier GII.17 circulated in 2013–2014 in RNA-dependent RNA polymerase (RdRp) (98.5%), lower in viral protein 2 (VP2) (97.7%) and lowest in VP1 (96.4%; Table 2). Genome scanning of 26 GII.17 Kawasaki 2014 strains against the prototype NS-405 revealed two variable regions, one in p22 of open reading frame 1 and another in VP1-interaction domain of VP2, although the nucleotide difference was only ∼3% (Supplementary Fig. 3). Pairwise nucleotide comparison on complete genome and genes (open reading frame 1, RdRp and VP2) with the prototype NS-405 suggested an overall increase in differences over time (*ρ* range, 0.38–0.67; *P*-value range, <0.001–0.06, Spearman's test; Supplementary Fig. 4). LOWESS data fitting on the RdRp showed a faster increase in nucleotide difference similar to that observed in VP1 starting from April 2015. Conservative MEME analysis inferred that codon 48 in p48 of open reading frame 1 was also under episodic diversifying selection (*P*=0.03, MEME test). No codons in RdRp and VP2 were found to be under positive selection.

### Broad saliva-binding profile of norovirus GII.17

**Figure 5:**
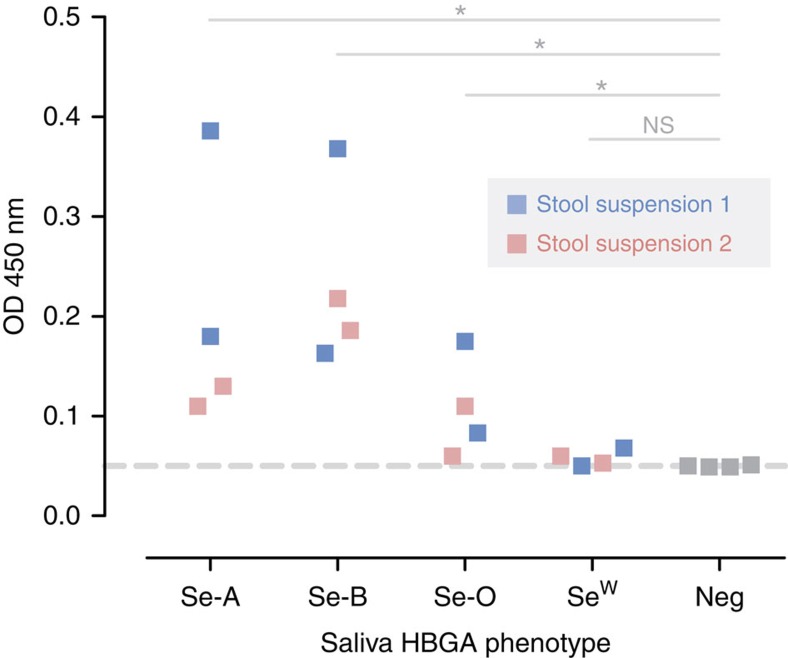
Broad saliva-binding profile of norovirus GII.17 Kawasaki 2014. Unfiltered stool suspensions were used. Virus binding was made using in-house saliva-coated wells, followed by norovirus detection with a commercial diagnostic kit. Testing was performed using saliva of two individuals for each secretor blood group A, B and O and weak secretor. Dashed horizontal line indicates lower detection limit of the assay. HBGA, histo-blood group antigen; Neg, negative controls; NS, not significant; OD, optical density; Se, secretor; Se^w^, weak secretor. *P<0.05 (Mann–Whitney *U*-test).

Stool suspensions of two representative GII.17 Kawasaki 2014 cases with VP1 sequence of the predominant strain (Fig. 2, purple circles) were tested for binding to saliva collected from two individuals for each histo-blood group/secretor status. Virus binding to secretor saliva of histo-blood groups A, B and less strongly to O was noted (all *P*<0.05 compared with negative controls, Mann–Whitney *U*-test); there was no statistically significant virus binding to saliva of weak secretors (Fig. 5).

### Sequential norovirus GII.4 and GII.17 infections

There were two case of sequential norovirus GII.4 and GII.17 infections. Both cases (a 4-year-old girl and a 2-year-old boy) were hospitalized for norovirus GII.17 Kawasaki 2014 in February 2015. Record review indicated another earlier episode of norovirus gastroenteritis attributed to GII.4 Sydney 2012 in the girl in February 2013 and in the boy in August 2014. Stool samples from both cases had a moderate level of noroviral loads (real-time reverse transcription–PCR (RT–PCR) cycle threshold values of 20–25).

## Discussion

In the 2014–2015 winter, rapid emergence and predominance of a previously rare norovirus genotype called GII.17 occurred in Hong Kong, which is a coastal city in southern China. This unusual increase in norovirus GII.17 activity during the same period was also reported in other Asian regions including China^14^,^15^ and Japan^16^. This GII.17 variant, first detected locally on 27 September 2014 (1–3 months ahead of other parts of China and Japan), was phylogenetically distinct from earlier GII.17 strains that circulated in 2013 and 2014. Here we provide strong evidence that the novel GII.17 Kawasaki 2014 is a fast-evolving, broadly recognizing and probably immune-escaped variant. Our findings offer important new understandings regarding norovirus epidemiology.

First, the novel GII.17 Kawasaki 2014 may have acquired epidemic capability. Molecular epidemiological studies have demonstrated that GII.4 was the major circulating norovirus genotype, accounting for 70–80% of norovirus outbreaks of various settings (for example, hospitals) and transmission routes (for example, person-to-person)^17^,^18^,^19^. Similar observations were reported locally^20^,^21^. Our studies in 2004–2005 and 2012–2013 also suggested that GII.4 was the most prevalent (∼90%) genotype in hospitalized norovirus gastroenteritis in Hong Kong^5^,^22^. In sharp contrast, norovirus GII.17 was rarely reported worldwide. This is exemplified by the huge difference in the volume of sequences deposited in GenBank. A simplified search using ‘norovirus GII.4' and ‘norovirus GII.17' returned 6,202 and 226 nucleotide entries as of September 2015, respectively. The only study that found GII.17 as the predominant norovirus genotype before the 2014–2015 winter was an environmental investigation of water sources during 2012–2013 in Kenya^23^. Interestingly, it appears that GII.17 cases were more commonly reported from studies conducted in Africa and Central/South America^24^,^25^,^26^. In a Paraguayan study of norovirus gastroenteritis, for example, both GII.4 and GII.17 had a similar prevalence of 18% in children aged ≤5 years^24^. The apparent characteristic geographical distribution may suggest GII.17 infections are dependent on host genetic factors. Human secretor status, which controls the expression of HBGA on intestinal epithelial cell surface, is one of the well-recognized factors that governs norovirus susceptibility^27^. Recently ancestry-based difference in secretor status is shown to influence norovirus epidemiology^28^,^29^. The very unusual rapid emergence and wide circulation of the novel GII.17 Kawasaki 2014 in Hong Kong and other Asian regions raises the concern that whether the new variant has overcome genetic barrier and has successfully explored evolutionarily advantageous HBGA binding. In norovirus GII, principally six residues (S343, T344, R345, D374, G442 and Y443; GII.4 numbering) on VP1 define the HBGA-binding interface^10^. In the VP1 P2 domain of the novel GII.17 Kawasaki 2014, there was one insertion at position 378 that corresponded to D374 of the HBGA-binding interface^15^,^16^. Limited by the absence of the crystal structure of GII.17 VP1 (ref. 30), the influence of the insertion on HBGA binding remains elusive. Nevertheless, our preliminary findings showed that GII.17 Kawasaki 2014 bound to saliva of secretors of various histo-blood groups. This binding profile appears to be as broad as that of several pandemic GII.4 variants^10^,^31^,^32^, implicating a substantial proportion of our population may be susceptible to infections of GII.17 Kawasaki 2014. This highlights the epidemic potential of this emergent novel variant.

Second, immune-escaped GII.17 Kawasaki 2014 targets all age groups and may cause severe gastroenteritis in previously less vulnerable population. To have a better chance of capturing emergent norovirus of substantial impact, we conducted our surveillance in hospitalized norovirus gastroenteritis that represents the severe end of the spectrum of disease. We reason that any change in the predominant circulating genotype or GII.4 variant in hospital cases may have crucial public health implications. Genotype change alone, however, may not be that surprising considering periodic high activity of norovirus non-GII.4 genotype is not uncommon^33^,^34^. Another strong reason that prompted us to initiate this detailed investigation was the wide age distribution of our GII.17 cases. Young children aged <5 years and adults aged >65 years (that is, those at the extremes of age) are known to be at-risk groups for more severe norovirus gastroenteritis partly due to immature and waning immunity, respectively^35^,^36^. Among our GII.4 cases, the median age was 1 year and at-risk age groups accounted for 81.0% of cases. This age distribution agreed well with the general norovirus epidemiology and clinical presentation that norovirus infection is mild (non-hospitalized) in otherwise healthy individuals. In sharp contrast, GII.17 Kawasaki 2014 targeted all age groups. The median age of our GII.17 cases was 49 years and the so-called at-risk groups only accounted for 52.3% of cases. Increased proportion of children and young adults aged 5–65 years is alarming because this previously less vulnerable age group is generally regarded as non-compromised, thus unlikely to develop severe norovirus gastroenteritis that required medical attention. Furthermore, higher proportion of elderly aged ≥85 years in GII.17 cases may have substantial clinical implications as this age group has been associated with most mortality in norovirus gastroenteritis^37^. Although we observed two cases of fatal GII.17 infections, direct causal relationship remains elusive. Detailed comparison of clinical features with norovirus GII.4 is underway to assess the virulence of this novel GII.17 Kawasaki 2014. One explanation for the wide age distribution is the virtually absence of immunity against the novel GII.17 Kawasaki 2014 across all age groups. This is possible because of the rarity of and thus lack of previous exposure to norovirus GII.17 until now. Moreover, residue substitutions and insertion occurred in four out of five inferred antigenic epitopes on the VP1 P2 domain, indicating the establishment of a probably immune-escaped variant. This may resemble the cyclic surge in norovirus GII.4 gastroenteritis that coincides with the epochal evolution of GII.4 variants through immune evasion^38^. Previous exposure to a different norovirus genotype, at least to GII.4 Sydney 2012, is likely to offer little or no cross-protection as suggested by our two cases of sequential (separated by 6 months to 2 years apart) GII.4 and GII.17 infections. The first known pandemic norovirus gastroenteritis occurred in 1996 with the emergence of the then novel GII.4 95/96 variant^39^, followed by the Farmington Hills variant in 2002 (ref. 40). The Farmington Hills variant and all other subsequent pandemic GII.4 variants differed from earlier GII.4 by a characteristic insertion at antigenic epitope D. Remarkably, the insertion at position 397 in the VP1 P2 domain of the novel GII.17 Kawasaki 2014 was also mapped to the inferred antigenic epitope D. Whether or not the insertion will confer pandemic potential to the novel GII.17 Kawasaki 2014 is of immense interest.

Third, recent GII.17 VP1 evolved faster than GII.4 VP1. Previous estimation using sequences dated back to 1974–2007 suggests the predominant norovirus GII.4 VP1 evolved at a steady rate of 4.3 × 10^−3^ nucleotide substitutions per site per year in the past four decades^11^. In our study, long-term estimation using root-to-tip regression and Bayesian BEAST analysis on strains spanning nearly 40 years as well as short-term estimation using LOWESS data fitting on strains from the 2014–2015 epidemic period all suggest rapid evolution of GII.17 VP1 at a rate of 1.2 × 10^−2^−2.1 × 10^−2^ nucleotide substitutions per site per year, which is one order of magnitude faster than that of GII.4 VP1 and other norovirus genotypes such as GII.3 and GII.7 (4.2 × 10^−3^−6.0 × 10^−3^ and 2.3 × 10^−3^ nucleotide substitutions per site per year, respectively)^6^,^11^,^12^,^13^,^41^. Root-to-tip analysis further hints that the evolutionary rate of norovirus GII.17 may not be constant and was accelerating in recent years. RNA viruses are well known to be fast-evolving at an overall rate of 10^−3^ nucleotide substitutions per site per year^42^ and the resultant genetic diversity is paramount to maintain viral fitness and survival under immune pressure^43^. Norovirus GII.4, the major circulating genotype since 1996 with epochal emergence of immune-escaped variants every 2–4 years^3^,^38^,^44^,^45^, was reported to have faster-evolving VP1 than other genotypes^41^, presumably explaining its predominance. We speculate that the intriguingly high evolutionary rate of GII.17 VP1 in recent years, probably through the acquisition of a novel RNA polymerase^16^,^46^, favour its host adaptation by fuelling the accumulation of residue substitutions on antigenic epitopes, driving antigenic drift. This consequently allows the probably immune-escaped novel GII.17 Kawasaki 2014 to, perhaps temporarily, outcompete the contemporary GII.4 Sydney 2012 that, after 2 years of its first emergence^47^, encountered herd immunity in the 2014–2015 winter. High frequency of mutations may also power GII.17 VP1 to explore evolutionarily advantageous broad HBGA binding. It is noteworthy that use of sequences collected over a short period of time may tend to over-estimating evolutionary rate^48^. Thus, far only 16 complete and near complete GII.17 VP1 sequences are available from GenBank over a 37-year-period (1978–2014) and all but 2 were collected after 2000. The paucity and temporal clustering of sequence data may lead to considerable uncertainty in estimating the evolutionary pattern and substitution rate of norovirus GII.17 VP1, as reflected in the wide 95% highest posterior density interval in spite of repeated attempts to boost the effective sample size parameter in Bayesian BEAST analysis. Rate overestimation over a very-short time frame (for example, within a few months) may also result from transient deleterious mutations commonly observed in RNA viruses^49^. Although not common, there were reports of sporadic norovirus GII.17 infections in several earlier epidemiological studies^17^,^26^,^50^,^51^. Complete sequencing of GII.17 VP1 from these cases to fill the data gap is highly anticipated to give us a better picture of GII.17 evolution in the past decades and a more robust estimation of its evolutionary rate.

Last, genome-wide analysis of norovirus GII.17 is proved challenging since only six complete genomes are available from the public domain as of this writing. Our study provides thus far the largest collection of 27 norovirus GII.17 genomes. Despite the limitation of short sampling time frame, preliminary analysis indicated that VP1-interaction domain of VP2, for example, is also rapidly mutating during the epidemic period, providing insights into norovirus evolution. Covariation of VP1 and VP2 in norovirus GII.4 variants has been suggested^52^. It would be very interesting to know whether such covariation is common among norovirus genotypes and whether VP2 plays a role in driving VP1 evolution apart from its structural functions^53^,^54^.

In conclusion, we report the epidemic spread of a novel norovirus GII.17 variant (Kawasaki 2014) that emerged during the 2014–2015 winter in Hong Kong. Multiple genetic signatures linked to HBGA binding and antigenic epitopes were identified. This fast-evolving, broadly recognizing and probably immune-escaped novel GII.17 Kawasaki 2014 causes severe gastroenteritis in previously less vulnerable population. Faster evolutionary rate of GII.17 VP1 than GII.4 VP1 reveals intense competition among norovirus genotypes and the potential of GII.17 to further acquire virulence and transmissibility. The predominance of a previously rare norovirus genotype clearly underlines the highly dynamic nature and the unpredictability of norovirus genotype circulation. It remains unclear whether GII.17 Kawasaki 2014 will persist in our community and demonstrate typical norovirus winter seasonality in the coming 2015–2016 winter. Considering norovirus GII.17 Kawasaki 2014 is still rapidly evolving after current epidemic spread and has been sporadically detected outside Asia^9^, close monitoring of the global spread of this novel GII.17 variant is necessary.

## Methods

### Study design and patients

An ongoing, systematic hospital-based local surveillance network has been established since August 2012 to detect new emergent norovirus GII.4 variants and to evaluate norovirus genotype distribution^47^. Findings from the surveillance focusing on viral loads of norovirus GII.4 have been published elsewhere^5^,^55^. This network consists of three major acute general hospitals in the Hong Kong Hospital Authority's New Territories East Cluster that has a catchment population of about 1.2 million. Patients with symptom of acute gastroenteritis were hospitalized if they presented with severe medical conditions considered unmanageable at home or if there were concerns over exacerbation of underlying illnesses. Stool samples were collected on admission and tested for norovirus upon clinical suspicion by using a real-time RT–PCR assay^56^. Laboratory diagnosis was performed at one hospital (Prince of Wales Hospital) in batches twice weekly. Positive cases were selected for further norovirus genotyping according to the following two-phase scheme: (i) in the pilot phase (August 2012–February 2014), all positive cases in the first test batch every week were genotyped; (ii) in the full surveillance phase (March 2014 onwards), all positive cases in both test batches every week were genotyped. Demographic information of age and sex and clinical information were retrieved from the computerized medical record system. Young children aged <5 years and adults aged >65 years were defined as at-risk groups for more severe norovirus gastroenteritis^35^,^36^. This study covered the full surveillance period from March 2014 to May 2015. Ethics approval for this study and waiver of informed written consent of using de-identified leftover routine diagnostic specimens were obtained from the joint Chinese University of Hong Kong-New Territories East Cluster clinical research ethics committee (reference numbers CRE-2013.330 and 2015.268).

### Norovirus genotyping

Noroviral RNA was extracted from ∼10% stool suspension using MagMAX Viral RNA Isolation Kit (Life Technologies). Purified viral RNA was first subjected to a GII.4-specific assay that amplified the complete VP1 gene using SuperScript III One-Step RT–PCR System (Life Technologies). Primers used were COG2F^56^ and NoV-Capsid-R (5′-GAGCCRAGGACATCAGATGCC-3′). Samples tested negative for GII.4 norovirus were subjected to a separate round of cDNA synthesis step and PCR detection as we described previously^52^. Briefly, viral RNA was converted into cDNA using a tagged oligo(dT) primer (5′-GACTGACTAGCTATCGGAGCATCG(T)_31_-3′) and SuperScript III reverse transcriptase (Life Technologies). PCR of an ∼2.7 kb fragment covering complete VP1, VP2 and 3′ untranslated region of the norovirus genome was performed using Phusion High-Fidelity DNA Polymerase (Thermo Scientific). Primers used were COG1F (for norovirus GI) or COG2F (for norovirus GII)^56^ and Tag (5′-GACTGACTAGCTATCGGAGCATCG-3′; for both norovirus genogroups). PCR amplicons were purified using either ExoSAP-IT (Affymetrix) or QIAquick PCR Purification Kit (Qiagen). Sequencing was performed using the forward PCR primer. Norovirus genotype assignment was based on the partial VP1 gene using the norovirus genotyping tool (http://www.rivm.nl/mpf/norovirus/typingtool).

### Complete norovirus GII.4 and GII.17 VP1 sequencing

Complete GII.4 and GII.17 VP1 sequences were determined by the primer walking approach using the 2.7-kb PCR amplicons generated above. Sequencing primers used were listed in Supplementary Table 3. DNA sequencing chromatograms were manually curated and contigs were generated using ChromasPro version 1.7.6 (Technelysium). Complete VP1 sequences obtained in this study have been deposited into GenBank under the following accession numbers: GII.17 (KP698928–KP698931, KP902563–KP902590 and KT315668–KT315719); GII.4 (KM268084–KM268107, KM396961, KM514057–KM514079, KM982935–KM982956, KP096327–KP096348, KP123606, KP176393–KP176412, KP241905–KP241915, KP293587–KP293591, KP698923–KP698927 and KT780371–KT780393).

### Norovirus GII.17 Kawasaki 2014 genome sequencing

Overlapping PCR amplicons, each of size ∼1 kb, spanning the complete norovirus genome were generated using tagged cDNA and Phusion High-Fidelity DNA Polymerase. PCR amplicons were directly sequenced using PCR primers. Sequence at the 5′-end was determined by using GeneRacer kit (Life Technologies). Primer sequences are available upon request. Complete/near complete (>99.4% coverage) genomes obtained in this study have been deposited into GenBank under accession numbers KP998539, KT326180–KT326182 and KT780394–KT780416.

### Nucleotide similarity analysis

All complete and near complete (>90% coverage) norovirus GII.17 VP1 nucleotide sequences (*n*=16) were downloaded from GenBank. Similarity plot was generated using SimPlot version 3.5.1 with a window size and step size of 200 and 20, respectively. Kimura 2-parameter distance model was used. The ratio of transitions and transversions was determined empirically.

### Homologous modelling

Three-dimensional structure of GII.17 VP1 was predicted by homologous modelling using SWISS-MODEL (http://swissmodel.expasy.org). The model was built on the basis of the crystal structure of the VP1 P domain of a GII.10 strain (PDB number 3V7A^57^) with default settings. Residue changes were mapped to the model. PyMOL version 1.5.0.3 was used for model visualization and colour annotation. Residue positions were numbered with reference to the novel GII.17 Kawasaki 2014.

### Evolutionary analysis

The best-fit nucleotide and amino-acid substitution models were determined using MEGA version 6.06. Models TN93+G (Tamura–Nei with a discrete gamma distribution) and JTT+G (Jones–Taylor–Thornton with a discrete gamma distribution) had the lowest (best) Bayesian Information Criterion scores for nucleotide and amino-acid analyses, respectively. Maximum likelihood phylogenetic inference rooted to non-GII.17 genotypes and the oldest complete GII.17 VP1 sequence available in GenBank (Hu/GII.17/C142/GF/1978, accession number KC597139) was then performed separately using the best-fit models. Nucleotide and amino-acid distances were calculated with reference to the oldest GII.17 strain listed above and plotted against sequences' year of collection. Path-O-Gen version 1.4 was used to perform root-to-tip regression analysis to estimate nucleotide substitution rates without a molecular clock assumption with reference to the oldest GII.17 strain listed above. Bayesian BEAST version 1.8.0 was used to estimate nucleotide substitution rate of norovirus GII.17 under the uncorrelated lognormal relaxed molecular clock model^6^. Briefly, substitution model GTR+I was used with empirical base frequency estimation and partition into three codon positions. Bayesian skyline coalescent model was used in tree prior calculation. Convergence of parameters was evaluated using Tracer version 1.6 and effective sample size value >200 was considered acceptable. Norovirus GII.4 was used as an outgroup. BEAST XML and trace files used were available upon request. To determine codons under episodic diversifying (positive) selection, complete coding sequences were tested using MEME method of HyPhy package implemented online on DataMonkey server (http://www.datamonkey.org) with a cutoff *P*-value of 0.05 as previously suggested^58^.

### Saliva-binding assay

Binding profile of the novel norovirus GII.17 Kawasaki 2014 to saliva was assessed using in-house saliva-coated wells in conjunction with a commercial norovirus ELISA detection kit. Briefly, stool suspension was first tested using RIDASCREEN Norovirus 3rd Generation kit (‘referred to as RIDASCREEN hereafter'; R-Biopharm). Samples tested positive were then subjected to saliva-binding assay. Archived saliva of six secretors (two individuals for each blood group A, B and O) and two weak secretors were used^59^. All saliva donors were adults of Chinese ethnic; secretor phenotype and genotype were confirmed by HBGA/Lewis antigens detection and *FUT2* 385 genotyping, respectively, in our previous study^59^. Each well of MaxiSorp ELISA pate (Nunc) was coated with 100 μl of boiled saliva supernatant (diluted 1:1,000 in 1 × phosphate-buffered saline) overnight. After 2 h blocking with 5% blotto (Pierce), wells were incubated with 100 μl of unfiltered virus-containing stool suspension (diluted 1:1 in RIDASCREEN's diluent). Detection was performed using RIDASCREEN as per manufacturer's instructions. Absorbance at 450 nm was measured. Wells were washed five times with RIDASCREEN's 1 × Wash Solution between steps. All steps were performed at room condition. Stool suspension from two individuals were used. Coated wells without addition of stool suspension and uncoated wells were used as negative controls.

### Statistical analysis

Categorical and continuous variables were presented as percentage and median with IQR, respectively. Categorical and continuous variables between two groups were compared using Fisher's exact test and Mann–Whitney *U*-test, respectively. Comparison of two continuous variables was performed using linear regression, Spearman's rank test or LOWESS fit line calculation where appropriate. A two-tailed *P*-value of <0.05 was considered statistically significant. All statistical calculations were performed using Prism version 5.04 for Windows (GraphPad) unless otherwise specified.

## Additional information

**Accession codes:** Nucleotide sequences obtained in this study have been deposited into GenBank under the following accession numbers: KM268084–KM268107, KM396961, KM514057–KM514079, KM982935–KM982956, KP096327–KP096348, KP123606, KP176393–KP176412, KP241905–KP241915, KP293587–KP293591, KP698923–KP698931, KP902563–KP902590, KP998539, KT315668–KT315719, KT326180–KT326182, KT780371–KT780416.

**How to cite this article:** Chan, M. C. W. *et al.* Rapid emergence and predominance of a broadly recognizing and fast-evolving norovirus GII.17 variant in late 2014. *Nat. Commun.* 6:10061 doi: 10.1038/ncomms10061 (2015).

## Supplementary Material

Supplementary Information Supplementary Figures 1-4 and Supplementary Tables 1-3

## Figures and Tables

**Table 1:** Codons of norovirus GII.17 VP1 under episodic diversifying selection.

Codon*	**VP1 domain**	***P*****-value**
354	P2	<0.001
371	P2	0.016

P2, protruding 2; VP1, viral protein 1.

^*^Novel GII.17 Kawasaki 2014 numbering.

**Table 2:** Percentage nucleotide identity between genomes of norovirus GII.17 Kawasaki 2014 and other GII.17 strains.

**Sequence name (GenBank accession number)**	ORF1*	**RdRp**	**VP1**	**VP2**
Hu/GII.P17_GII.17/Kawasaki323/JP/2014 (AB983218)	98.1	98.5	96.4	97.7
Hu/GII.P16_GII.17/Saitama/T87/JP/2002 (KJ196286)	76.5	78.1	76.7	74.3
Hu/GII.17/C142/GF/1978 (KC597139)	76.9	79.2	77.0	74.2

ORF1, open reading frame 1; RdRp, RNA-dependent RNA polymerase; VP1, viral protein 1; VP2, viral protein 2.

^*^Excluding RdRp.
