# Supplementary material for: Rapid emergence and predominance of a broadly recognizing and fast-evolving norovirus GII.17 variant in late 2014
Source: Nat Commun. 2015 Dec 2;6:10061. doi: 10.1038/ncomms10061 (PMC4686777; doi:10.1038/ncomms10061)
Supplement: Supplementary Information — Supplementary Figures 1-4 and Supplementary Tables 1-3 [file ncomms10061-s1.pdf]

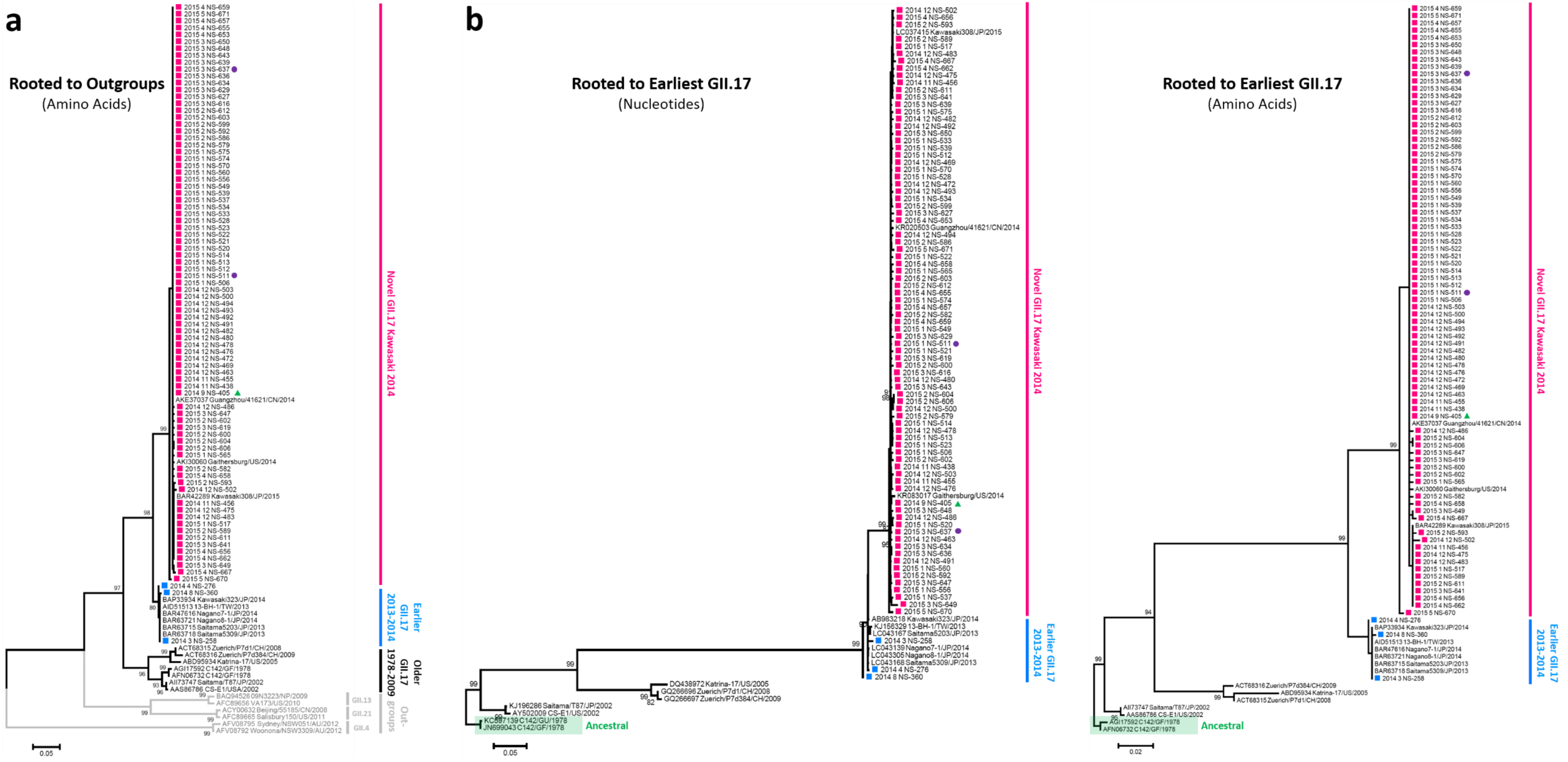

Supplementary Figure 1 | Emergence of norovirus GII.17 Kawasaki 2014 during the 2014/15 winter in Hong Kong. Maximum likelihood phylogenetic trees of complete and near complete viral protein 1 nucleotide and amino acid sequences were rooted to **(a)** non-GII.17 genotypes and **(b)** the oldest GII.17 strains. Gaps in alignment were neglected in the analysis and the final dataset contained 1,586 nucleotide and 526 amino acid positions. Shown are trees with the highest log likelihood. Statistical evaluation was performed by 1,000 bootstrap replications and percentages of clustering (≥80%) are shown at nodes. Magenta and blue squares denote GII.17 Kawasaki 2014 and earlier GII.17 sequences obtained in this study, respectively. Green triangle denotes the first case of GII.17 Kawasaki 2014 in Hong Kong. Purple circles indicate cases selected for saliva binding assay. Scale bars indicate the number of substitutions per site. Sequence nomenclature: year-month of collection followed by unique sequence identifier (for GII.17 sequences acquired in this study); GenBank accession number followed by unique sequence identifier, country of origin, and year of collection (for GII.17 sequences downloaded from GenBank). Country/region abbreviations: AU, Australia; CH, Switzerland; CN, China; GF, French Guiana; JP, Japan; NP, Nepal; TW, Taiwan; US, the United States of America.

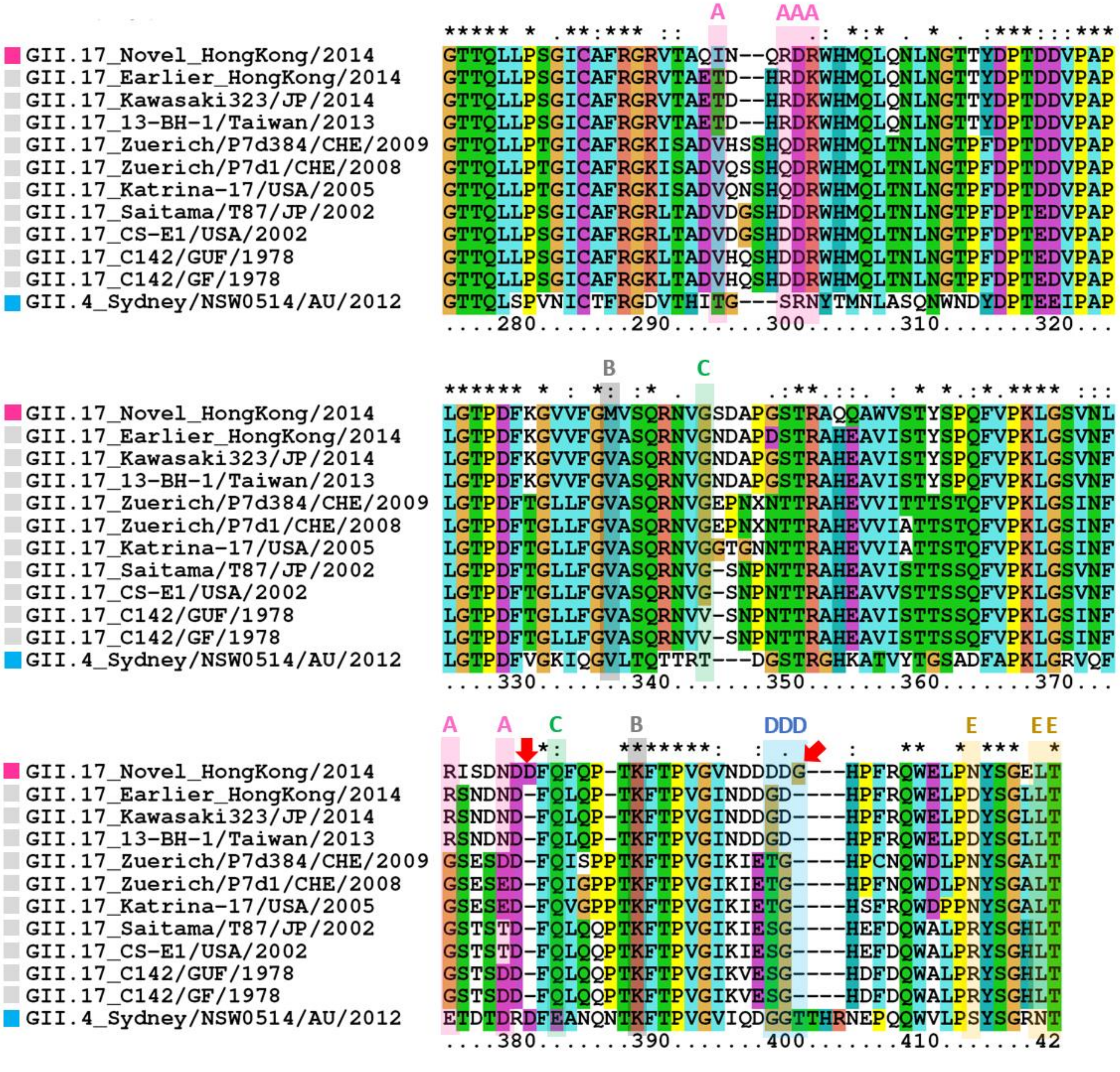

Supplementary Figure 2 | Multiple amino acid sequence alignment of norovirus GII.17 VP1 P2 domain. Positions of GII.4 antigenic epitopes A—E (as defined in reference[60]) are shaded in different colors and labelled at the top. Magenta and blue squares at the leftmost side denote the novel GII.17 Kawasaki 2014 and GII.4 Sydney 2012, respectively. Red arrows indicate amino acid insertions exclusively found in the novel GII.17 Kawasaki 2014. P2 domain was defined as residue positions 274—419 (GII.4 numbering).

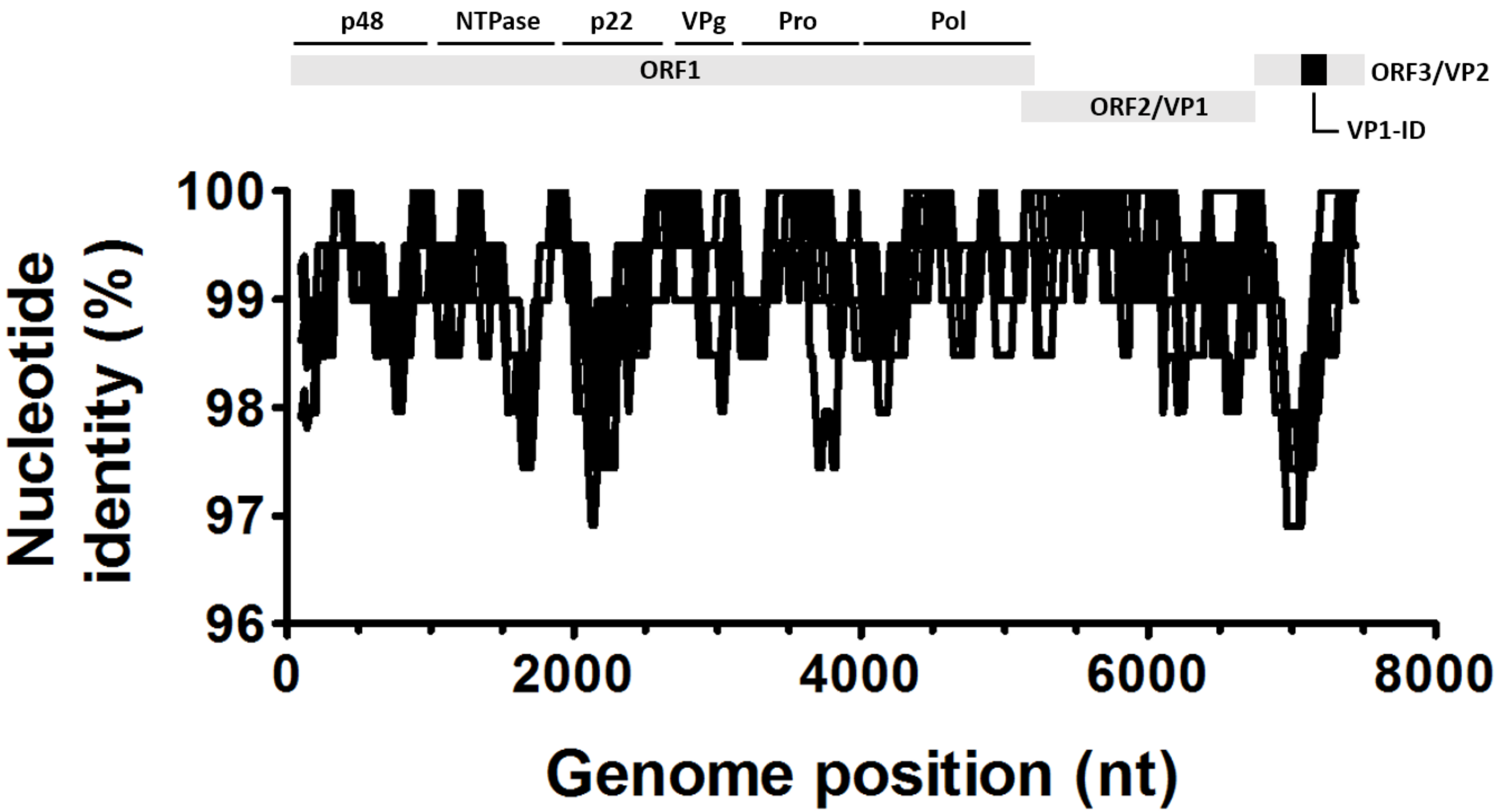

Supplementary Figure 3 | Genome scanning of norovirus GII.17 Kawasaki 2014. SimPlot analysis was performed on 27 complete/near complete genomes of GII.17 Kawasaki 2014 collected at different months against the prototype strain, NS-405, collected on 27 September 2014. Schematic representation of norovirus genome organization was not drawn in scale. Abbreviations: nt, nucleotide; ORF, open reading frame; Pol, RNA-dependent RNA polymerase; Pro, protease; VP1, viral protein 1; VP1-ID, VP1-interaction domain; VP2, viral protein 2; VPg, viral protein genome-linked.

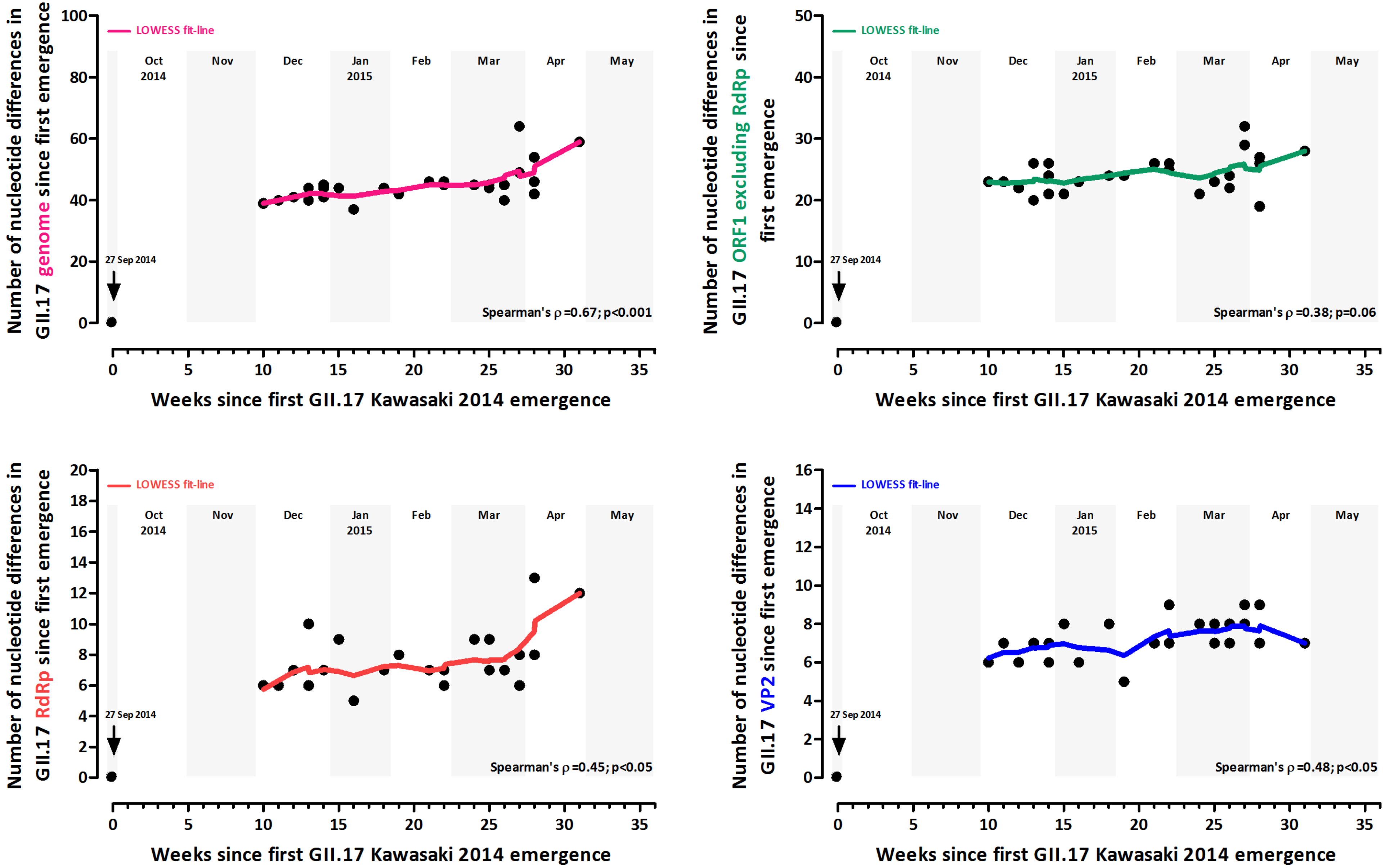

Supplementary Figure 4 | Complete genome analysis of norovirus GII.17 Kawasaki 2014. Shown are numbers of accumulated nucleotide differences of local GII.17 Kawasaki 2014 compared with the first strain NS-405 (black arrow) collected on 27 September 2014 in Hong Kong against time (in weeks) since first emergence, grouped by open reading frames (ORFs) and genes. NS-405 was excluded in LOWESS data fitting and Spearman's correlation calculation. Colored lines denote LOWESS fit-lines. RdRp, RNA-dependent RNA polymerase; VP2, viral protein 2.

Supplementary Table 1 | Number of norovirus GII.17 VP1 and genome sequenced in each month.

**Year**	**2014**										**2015**				
**Month**	**3**	**4**	**5**	**6**	**7**	**8**	**9**	**10**	**11**	**12**	**1**	**2**	**3**	**4**	**5**
Total number of GII.17 detected	1	1	0	0	0	1	1	0	3	26	39	23	22	8	3
Number of GII.17 VP1 sequenced, n (%)	1 (100)	1 (100)	0	0	0	1 (100)	1 (100)	0	3 (100)	17 (65)	22 (56)	14 (61)	14 (64)	8 (100)	2 (67)
Number of GII.17 genome sequenced, n (%)	0 (0)	0 (0)	0 (0)	0 (0)	0 (0)	0 (0)	1 (100)	0 (0)	0 (0)	7 (27)	4 (10)	4 (17)	6 (27)	5 (63)	0 (0)

Supplementary Table 2 | Number of norovirus GII.4 Sydney 2012 VP1 sequenced in each month.

**Year**	**2014**										**2015**				
**Month**	**3**	**4**	**5**	**6**	**7**	**8**	**9**	**10**	**11**	**12**	**1**	**2**	**3**	**4**	**5**
Total number of GII.4 Sydney 2012 detected	3	6	5	11	23	28	22	14	14	10	17	2	2	4	0
Number of GII.4 Sydney 2012 VP1 sequenced, n (%)	3 (100)	6 (100)	5 (100)	11 (100)	22 (96)	28 (100)	22 (100)	14 (100)	13 (93)	8 (80)	15 (88)	2 (100)	2 (100)	4 (100)	0 (0)

Supplementary Table 3 | Primers used in norovirus GII.4 and GII.17 VP1 sequencing.

Primer Name	Primer Sequence (5' to 3')	Norovirus Genotype
NoV-GII.17-VP1-F2	GTGATGATGTCTTTACTGTCTCTTGCA	GII.17
NoV-GII.17-VP1-F3	AAATTCACACCAGTKGGMATCAA	GII.17
NoV-GII.4-VP1-R2	CCTAACATCRGGTAAGGGRATCA	GII.4
NoV-GII.4-VP1-F1.5	ACAGTATCCCCTAGAAACGCTCCA	GII.4
NoV-GII.4-VP1-R	GAGCCRAGGACATCAGATGCC	GII.4
